# Anxiety and Depression and Sleep Problems Among Patients with Fetal Alcohol Spectrum Disorders

**DOI:** 10.3390/children12010001

**Published:** 2024-12-24

**Authors:** Katarzyna Anna Dyląg, Wiktoria Wieczorek-Stawińska, Agata Cichoń-Chojnacka, Anna Piaskowska, Katarzyna Jaroszewska, Kornelia Wasiak, Tomasz Jagła, Julia Frączek, Aneta Abram, Adriana Costanzo, Federica Landri, Paulina Dumnicka, Lech Popiołek

**Affiliations:** 1 Department of Pathophysiology, Jagiellonian University Medical College, Świetej Anny 12, 31-008 Krakow, Poland; katarzyna.anna.dylag@uj.edu.pl; 2 St. Louis Children Hospital, Strzelecka 2, 31-503 Krakow, Poland; 3 Gdynia FASD Diagnosis and Therapy Center, Morska 112b/111, 81-225 Gdynia, Poland; 4 Department of Medical Biochemistry, Jagiellonian University Medical College, Mikołaja Kopernika 7C, 31-034 Krakow, Poland; 5 Ignatianum University in Cracow, Institute of Psychology, Sleep Research Laboratory, Mikołaja Kopernika 26, 31-501 Krakow, Poland

**Keywords:** fetal alcohol spectrum disorder, prenatal alcohol exposure, sleep disturbances, depression, anxiety

## Abstract

**Background/Objectives**: Sleep disturbances are common among children with fetal alcohol spectrum disorders (FASD) and are often accompanied by emotional and behavioral challenges. This study aimed to evaluate the relationship between sleep problems, anxiety, and depressive symptoms in children with FASD. **Methods**: The study included 90 children aged 7 to 16 years diagnosed with FASD, who were primarily in foster or adoptive care. Participants completed validated psychometric tools, including the Children’s Sleep Habits Questionnaire (CSHQ), State Trait Anxiety Inventory for Children (STAIC) and Children’s Depression Inventory 2 (CDI 2). **Results**: Sleep disturbances were significant, with 71.1% of participants scoring above the clinical threshold in CSHQ. State anxiety, measured by STAIC C-1, was positively correlated with specific sleep difficulties, such as bedtime resistance (r = 0.30, *p* = 0.008) and sleep anxiety (r = 0.31, *p* = 0.005). However, no correlation was found between trait anxiety (STAIC C-2) and sleep problems. Parent-reported depressive symptoms, measured using CDI 2:P, were strongly associated with general sleep disturbances (r = 0.27, *p* < 0.011), parasomnias (r = 0.33, *p* = 0.002) and daytime sleepiness (r = 0.34, *p* < 0.001). **Conclusions**: These findings suggest that sleep disturbances in children with FASD are closely related to state anxiety and depressive symptoms. The results emphasize the need for targeted interventions addressing sleep and emotional health in this population. Further research is needed to examine these relationships and their implications for clinical practice.

## 1. Introduction

Sleep issues are a common concern among children with neurodevelopmental disorders (NDs) [1,2]. Sleep disturbances and circadian rhythm alterations interfere with the clinical picture, contributing to attention deficits and cognitive and behavioral complaints [3,4].

Fetal alcohol spectrum disorders (FASDs) are a group of conditions resulting from prenatal alcohol exposure (PAE), and are considered the most prevalent neurodevelopmental disabilities in industrialized countries. Throughout this spectrum, the most severe presentation is Fetal Alcohol Syndrome (FAS), which is characterized by growth retardation, specific facial deformities and central nervous system (CNS) dysfunction. However, the FASD spectrum includes a wide range of symptoms, from mild cognitive impairments to severe behavioral and intellectual dysfunctions. Children with FASD often exhibit difficulties in concentration, memory, problem-solving and establishing social relationships, and these problems increase with age. The absence of distinct physical features does not exclude significant neurocognitive and behavioral deficits [5].

The worldwide prevalence of FASD is estimated to be 7.7 to 22.7 cases per 1000 individuals [6,7]. The symptomatology of the disease is mainly due to the toxic effects of PAE on the CNS and results in permanent structural and functional changes in the brain. Areas such as the corpus callosum, hippocampus, cerebral cortex, caudate nucleus and cerebellum are particularly affected, resulting in problems with memory, attention, emotion and executive functions [8,9,10,11]. Alcohol disrupts critical brain development processes such as neuronal migration, synaptogenesis and myelination, resulting in a reduction in the volume of white and gray matter [12]. These effects depend on the amount of alcohol consumed and the stage of pregnancy, with the highest risk during the first trimester. However, it still remains unclear exactly how much alcohol it takes, and at which precise stage of pregnancy, to cause specific changes in the fetal nervous system [13].

Although the general intelligence of individuals with FASD is generally within the norm [14,15], the patients usually present with other cognitive issues such as executive functioning deficits [16,17,18,19], speech delay [20,21], attention problems or specific learning disabilities [22,23,24]. Moreover, social, emotional and behavioral symptoms occur frequently in this group of patients [19,25,26,27,28,29,30,31]. It has been established that impaired adaptive functioning [23,32,33,34] and self-regulation skills are also a major concern among patients with FASD. Executive functioning deficits are among the most prevalent issues in individuals with FASD, involving difficulties with planning, organization, problem-solving and regulating impulsive behaviors, which profoundly affect daily life [16,17,18]. Speech and language delays, including difficulties in expressive and receptive language, further hinder interpersonal communication and often lead to frustration [20,21]. Attention problems and specific learning disabilities, such as challenges in reading, writing, and mathematics, increase the risk of academic underperformance, necessitating tailored educational approaches [22,23,24]. Impaired adaptive functioning and self-regulation skills significantly affect individuals’ ability to cope with changing circumstances, manage frustration, and control emotional responses. These deficits can lead to impulsive behaviors and interpersonal conflicts, complicating independent functioning in adulthood, including maintaining employment and forming lasting relationships [32,33,34]. Social, emotional and behavioral symptoms are highly prevalent, including anxiety, depression, difficulties in peer relationships and risky behaviors [35].

Studies confirm that alcohol-induced damage is a primary cause of these cognitive and behavioral issues, highlighting the importance of early diagnosis and intervention to improve outcomes and prevent secondary psychosocial complications.

Both subjective questionnaire studies [36,37,38,39] and objective tools such as polysomnography or actigraphy [40,41,42] have confirmed that among patients with FASD, sleep disorders are highly prevalent. The general frequency of sleep disorders is estimated to be 55–85% [37,43,44]. It has been established that children with FASD have problems with sleep-onset delay, parasomnias, night wakings and sleep disordered breathing. Although the increased prevalence of sleep disorders has been established, the reciprocal relationship between sleep and neurobehavioral syndromes has gained little consideration. O’Rourke at al. indicated that sleep problems are independent of neurobehavioral characteristics of children with FASD [45]. In contrast to these findings, Mughal et al. indicated the relationship between sleep and cognition in this group of patients [46]. The same research group suggested that, similarly to those typically developing peers, among patients with FASD, sleep problems correlate with nightmares and generalized anxiety [42,47]. Benson et al. documented both increased anxiety and a significant tendency to sleep problems among patients with FASD; however, they have not attempted to correlate these factors [48]. It has been documented that sleep problems and anxiety are associated among both typically developing children and children with neurodevelopmental problems [48]. A relationship between sleep and depression and anxiety is bidirectional [49,50].

There is a gap in the knowledge regarding the emotional and cognitive characteristics of patients with FASD with sleep disorders. The objective of this study was to investigate the association between sleep problems and depression and anxiety among patients with FASD.

## 2. Materials and Methods

### 2.1. Study Group

The study group was recruited in two FASD diagnostic centers: one in a public regional hospital in Krakow, Poland, and another in a community center in Gdynia, Poland. All consecutive patients were offered participation in the study. All patients were diagnosed according to Polish diagnostic criteria [51]. According to the Polish guidelines, each patient has to be evaluated by a physician and a psychologist. Based on a dysmorphic features examination and neuropsychological examination, three diagnoses are made as follows: fetal alcohol syndrome (FAS), neurodevelopmental disorder with prenatal alcohol exposure (ND-PAE) or “risk group for FASD”. For each participant, the diagnosis was made prior to the start of the project by an interdisciplinary team consisting of a pediatrician, a psychiatrist and a psychologist. The exclusion criterion for the study was the presence of coexisting lung disease that could interfere with the breathing process during sleep. After obtaining informed consent, the study participants and their guardians were asked to complete a set of questionnaires. All psychological assessments were conducted by licensed psychologists. None of the patients were treated with benzodiazepines, selective serotonin reuptake inhibitors (SSRI), monoamine oxidase inhibitors (MAOi) or melatonin.

### 2.2. Psychometric Tools

The following psychometric tools were used during the study:

1. Children’s Sleep Habits Questionnaire (CSHQ), developed by Owens, Spirito and McGuinn [52];
2. State–Trait Anxiety Inventory for Children (STAIC), created by a team comprising C.D. Spielberger, C.D. Edwards, R.E. Lushene, J. Montouri and D. Platzek [53];
3. Children’s Depression Inventory 2 (CDI 2), authored by M. Kovacs [54].

#### 2.2.1. Children’s Sleep Habits Questionnaire

The Children’s Sleep Habits Questionnaire (CSHQ) consists of 33 questions about sleep-related habits and sleep disturbances present in the child. This tool assesses eight different domains: bedtime resistance, sleep-onset delay, sleep duration, sleep anxiety, night wakings, parasomnias, sleep-distorted breathing and daytime sleepiness [52]. The responses to individual questions are typically provided by the child’s parents and are then rated on a 3-point scale (ranging from “usually” to “sometimes” to “rarely”). This questionnaire has demonstrated acceptable psychometric properties not only in its English version but also in other language versions [55,56,57]. Consequently, it has been widely used to assess sleep in various populations of healthy children [58,59], as well as in children with health conditions [60,61].

Studies conducted both in a community sample and among children diagnosed with sleep disorders indicate that the reliability of the various CSHQ scales differs significantly. The Cronbach’s alpha coefficients for each scale range as follows: 0.70–0.83 for Bedtime Resistance, 0.69–0.80 for Sleep Duration, 0.63–0.68 for Sleep Anxiety, 0.44–0.54 for Night Wakings, 0.36–0.56 for Parasomnias, 0.51–0.93 for Sleep-Disordered Breathing and 0.65–0.70 for Daytime Sleepiness. Overall, the CSHQ demonstrates an internal consistency of 0.63 in the community sample and 0.78 in a clinical sample of children with diagnosed sleep disorders [52].

#### 2.2.2. State–Trait Anxiety Inventory for Children

The STAIC (State–Trait Anxiety Inventory for Children) is a psychometric tool designed to assess anxiety levels in children and adolescents. This method consists of two scales: one measuring state anxiety (Scale C-1) and the other measuring trait anxiety (Scale C-2). Scale C-1 evaluates the anxiety experienced at a specific time, while Scale C-2 assesses a child’s general predisposition to feel anxious in various life situations. Each scale comprises 20 statements to which the respondent can respond using a 3-point scale. Both scales demonstrate high internal consistency and strong theoretical validity confirmed through numerous studies. The STAIC inventory has been translated into multiple languages [52,62,63] and has been used in research involving children with various somatic illnesses [64,65] as well as those with mental disorders.

The STAIC questionnaire was translated into Polish by Sosnowski, Iwaniszczuk and Spielberger in 1987. Normalization and validation studies were carried out in 2004 on a group of 2224 Polish children and adolescents of both sexes [66]. The reliability of the scales was evaluated using Cronbach’s alpha internal consistency coefficients. In the normalization studies, these coefficients ranged from 0.87 to 0.91 for the C-1 scale and from 0.86 to 0.89 for the C-2 scale [66].

#### 2.2.3. Children’s Depression Inventory 2

The set of CDI 2 questionnaires is used to assess the severity of depressive symptoms in children and adolescents. It is widely used in clinical practice in various countries and often helps diagnose mood disorders in individuals under 18 years of age [67,68]. The set comprises several separate tools: a self-report questionnaire (available in full and short versions), a parent-report questionnaire, and a teacher questionnaire. In this study, the short version of the self-report questionnaire (CDI 2: SR(S)), which contains 12 items, and the parent questionnaire (CDI 2: P), which consists of 17 items, were used. The first of these questionnaires allows for a general estimate of the severity of depressive symptoms. The second enables a broader assessment of the child’s functioning. It can be used to assess the severity of depressive symptoms, as well as emotional and functional problems. The questionnaires included in the CDI 2 set are characterized by high Cronbach alpha coefficients and have confirmed discriminant validity in various studies.

Between 2013 and 2014, Polish researchers conducted normalization studies on the CDI 2 questionnaire set. The normalization sample for the short self-report version (CDI 2:SR(S)) consisted of 1010 boys and girls aged 7 to 18. Similarly, the normalization studies for the parent-report version (CDI 2:P) were based on data from 1018 participants. The reliability of the CDI 2:SR(S) TS scale, as measured by Cronbach’s alpha, ranged from 0.73 to 0.80. For the parent-report version, Cronbach’s alpha coefficients fell between 0.82 and 0.86 for the CDI 2:P TS scale, between 0.76 and 0.82 for the CDI 2:P EP scale, and between 0.69 and 0.76 for the CDI 2:P FP scale [69].

### 2.3. Statistical Analysis

Data collected were analyzed using the Statistica 13.3 PL statistical package (TIBCO Software Inc., Palo Alto, CA, USA), with a significance level of α = 0.05. In the initial stage of the analysis, the distribution of continuous variables was assessed using the Shapiro–Wilk test. Variables with distributions that did not significantly deviate from normality were compared using Student’s *t* tests. For variables with distributions significantly different from normality, Mann–Whitney U tests were applied. Correlation analyses were conducted using Spearman rank correlation coefficients.

### 2.4. Ethical Considerations

All study participants and their legal guardians gave their informed consent to participate in the project. The study was conducted anonymously and each participant was informed that they could withdraw their consent at any time without providing a reason. The study protocol was approved by the regional ethics committee of the Regional Board of Physicians (approval number: 162/KBL/OIL/2023). The research was carried out according to the ethical standards set forth by The World Medical Association (Declaration of Helsinki).

## 3. Results

### 3.1. Subjects

Our sample included 90 individuals (49 boys and 41 girls) who met the diagnostic criteria for fetal alcohol spectrum disorders. The median age of the participants was 10.5 years, with an interquartile range (IQR) of 3 years. The youngest participant was 7 years old and the oldest was 16 years old. More than half of the participants (51.1%) were raised in adoptive families; approximately one-quarter (25.6%) lived in foster families, 12 participants (13.3%) resided in institutions, and only 10% were raised by their biological parents.

### 3.2. Psychological Evaluation

Psychological evaluations revealed elevated levels of anxiety and trait depressive symptoms, particularly as reported by parents (Table 1).

Sleep habits in the study group were assessed using the CSHQ, with valid data from all 90 participants. The median total sleep disturbance score was 45 points (IQR: 41–50). In particular, 64 participants (71.1%) had a total sleep disturbance score greater than 41, which is indicative of clinically significant sleep problems. Additionally, the total score of the CSHQ was equal to 41 in 11 children (12.2%). The median scores for the CSHQ subscales are indicated in Table 2.

Table 3 shows that significant positive correlations were observed between the raw STAIC C-1 scores, which measure state anxiety, and both bedtime resistance (r = 0.30, *p* = 0.008) and sleep anxiety (r = 0.31, *p* = 0.005). This indicates that higher levels of state anxiety are associated with greater difficulties in initiating sleep and greater anxiety surrounding sleep in children. No significant correlation was found between the raw STAIC C-1 scores and the total CSHQ score. When analyzing STAIC C-1 centile scores, correlations with specific sleep difficulties remained significant. Furthermore, STAIC C-1 centiles were significantly correlated with the total CSHQ score (r = 0.24; *p* = 0.031), indicating that higher state anxiety may be related to overall poorer sleep habits in children and adolescents.

Table 4 summarizes the significant correlations between the Children’s Depression Inventory 2 (CDI 2) scores and the Children’s Sleep Habits Questionnaire (CSHQ) subscales. Higher total depression scores reported by parents (CDI 2: P TS) were significantly associated with increased daytime sleepiness (r = 0.32 to 0.34, *p* < 0.002) and general sleep disturbances (r = 0.27, *p* < 0.011). Emotional problems reported by parents (CDI 2: P EP) showed positive correlations with parasomnias, daytime sleepiness and the total CSHQ score (r = 0.33 to 0.37, *p* < 0.002), indicating that emotional issues are related to sleep difficulties in children. Interestingly, self-reported depression scores (CDI 2:SR(S) TS) demonstrated a significant negative correlation with parasomnia (r = −0.22 to −0.26, *p* < 0.037), suggesting complex interactions between self-perceived depressive symptoms in children and sleep disturbances.

**Figure 1:**
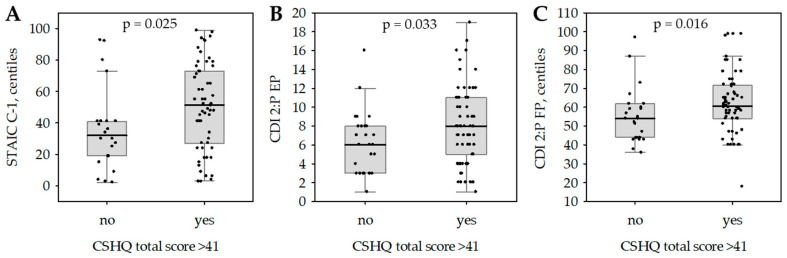
(**A**) Differences in STAIC C-2 centiles between groups with and without sleep disorders. (**B**) Differences in cdi 2: P EP between groups with and without sleep disorders. (**C**) Differences in CDI 2: P EP centiles between groups with and without sleep disorders. Statistically significant differences between children with high and low results on CSHQ. Data are shown as median (central line), interquartile range (box), non-outlier range (whiskers) and raw data (points); *p*-values calculated in the Mann–Whitney test are given. CDI 2: P—Children’s Depression Inventory 2: Parents; CSHQ—Children’s Sleep Habits Questionnaire; EP—Emotional Problems; FP—Functional Problems; STAIC—State–Trait Anxiety Inventory for Children.

Figure 1 compares children with and without behavioral and medically based sleep problems, defined by a total score on the Children’s Sleep Habits Questionnaire greater than 41. The analysis, using the Mann–Whitney U Test, revealed that children with sleep problems exhibited significantly higher levels of state anxiety and depressive symptoms. Specifically, children with sleep problems scored higher on state anxiety (STAIC C-1 centiles), with a median score of 51.5 compared to 32 for children without sleep problems (*p* = 0.025). This means that they feel more anxious in the present moment. Parents also reported more emotional problems (CDI 2: P EP) in children with sleep disturbances, with median scores of eight versus six in the group without sleep problems (*p* = 0.033). Additionally, functional problems (CDI 2: P FP centiles), which are difficulties in daily activities, were higher in the sleep problem group, with a median score of 60.5 compared to 54 (*p* = 0.016).

## 4. Discussion

We observed an increased prevalence of sleep disorders in children studied with FASD. Seventy-one percent of the patients had CSHQ indicating sleep disturbances (>41 points) out of which only 12% scored a borderline number (41 points). Furthermore, the median score in the group was high enough to indicate sleep disturbances (45 points (41; 50)). In the previous study of our group, we indicated a lower number of 55% [43]. The current result is more consistent with the results published by Chen et al., who suggested a prevalence rate as high as 85% [44]. Interestingly, all of the aforementioned studies were performed using the same questionnaire with established sensitivity and specificity; however, in our current investigation, the highest number of patients was enrolled.

We document an increased tendency that sleep disorders (CSHQ > 41) are associated with state anxiety (reflected in STAI C-1 centiles). We have not documented a correlation between trait anxiety (STAI C-2) and sleep disorders. Previous findings correlated sleep duration and sleep complaints with trait anxiety among adults and adolescents [70,71]. However, a general score of trait anxiety in our sample was increased (71 centile (49; 88)), what could indicate that a tendency to anxious reactions is one of the characteristics of patients with FASD, independently of sleep disorders. Indeed, both biological [72,73,74] and psychosocial [75,76,77,78] predictors of anxiety apply to this group. Therefore, only state anxiety might cause an increased occurrence of sleep problems. To our knowledge, this is the only report in which state/trait anxiety was measured in the context of sleep problems in FASD. Other authors focused on DSM-5 diagnoses indicating anxiety, therefore the comparison is challenging [79]. Mughal et al. established that sleep disturbances predicted a tendency to panic, separation anxiety, physical injury fears, and social phobia, but they have not documented a correlation between generalized anxiety and sleep problems [42]. The same correlations were observed in a group of typically developing children [42]. The same group of researchers documented an association between anxiety and nightmares [47]. Interestingly, in children without neurodevelopmental disabilities generalized anxiety was associated with the frequency of sleep problems [80]. On the other hand, Benson and colleagues established an increased tendency to both sleep problems and anxiety among children with FASD but did not perform a correlation analysis between these two areas [48]. In our report we document a significant correlation between depressive symptoms measured by CDI and the severity of sleep problems measured by the CHSQ score. We observed significant correlations for CDI total score, functional and emotional problems. Interestingly, these observations were significant for parental but not for child reports. These findings might be due to the fact that caregivers of patients with FASD experience significant distress [81,82,83] and might overreport some complaints. Furthermore, the psychologists who conducted the study suggested that children might have misinterpreted some of the questions and that their reports might be less reliable than their parents’ reports. This may be due to cognitive [19,28] or speech-related [20,21] problems that occur in FASD. Therefore, we indicate that sleep problems are associated with depressive symptoms among patients with FASD. To our knowledge, this is the first study to document this relationship. Evidence linking sleep problems with neurobehavioral symptoms is scarce. Hayes et al. established that sleep problems are associated with more behavioral symptoms among patients with FASD [39]. Mughal et al. indicated a relationship between sleep disturbances and cognitive functions such as working memory, reaction time, non-verbal tasks, and receptive language [46]. Furthermore, Chandler-Mather suggested that sleep disturbances affect executive functions, the domain that contributes to the most difficulties observed among patients with FASD [84]. All the aforementioned authors agree that sleep problems are a part of clinical picture in FASD, they should be evaluated during clinical visits and more research is needed to determine precise relationships between selected brain domains and sleep [39,46,47,48,85].

There is abundant evidence on associations between anxiety and neurobehavioral conditions [86,87,88,89,90]. In our study, we documented an increased tendency to trait anxiety in the FASD group. Anxiety is a common complaint of people with FASD [91,92] and disorders of anxiety are considered a frequent comorbidity of FASD [93]. Research on animal models of FASD also indicated an increased susceptibility to anxious behavior associated with prenatal alcohol exposure [74,94,95]. Senturias et al. established another premise that anxiety is a major concern among patients with FASD, documenting the significant use of antianxiety medications in this group of patients [96]. Benson et al. established increased scores for anxiety in Child Behavior Checklist (CBC) and increased scores for almost every domain in dedicated tool-Spence Children’s Anxiety Scale (SCAS) [48]. Mughal documented higher scores for separation anxiety, panic, generalized anxiety, social phobia, physical injury phobia and obsessive–compulsive disorder [42]. A developmental predisposition to depression resulting from PAE was documented by Hellemans et al. [94]. Benson et al. established a higher depression/anxiety score in CBCL than among healthy controls [48]. To our knowledge, there are no studies that evaluated depression among patients with FASD with a dedicated tool. Interestingly, Flanningan noted that both depression and anxiety tend to affect more women than men with FASD [97].

Sleep plays a crucial role in mental health, disruptions in sleep architecture are frequently linked to anxiety and depression. These relationships are bidirectional: poor sleep exacerbates symptoms of anxiety and depression, while these conditions further alter sleep regulation. The neurobiological mechanisms underlying this interaction involve complex systems, including the hypothalamic-pituitary-adrenal (HPA) axis, which is critical for stress regulation [98,99]. Anxiety disorders are often accompanied by hyperarousal, which makes it difficult to initiate and maintain sleep. On the other hand, chronic sleep disorders worsen anxiety symptoms by altering emotional processing and stress resilience [49]. In the same way, sleep problems, such as insomnia and hypersomnia, are closely linked to depression and can be seen as both risk factors and symptoms of the disease. REM sleep disturbances reduced slow-wave sleep and altered sleep-wake cycles observed in depression can all contribute to mood dysregulation and cognitive impairments [100]. The HPA axis mediates the effects of stress on sleep. Under normal circumstances, the HPA axis regulates the circadian rhythm of cortisol secretion, allowing for a proper sleep-wake cycle [101]. However, in individuals with anxiety or depression, dysregulation of the HPA axis often results in hypercortisolemia and increased stress responses. This overactivation disrupts sleep by increasing nightly arousal and interferes with the stability of sleep phases [102]. In anxiety disorders, HPA axis overactivity contributes to increased alertness and decreased sleep efficiency. Persistent activation of this axis increases the synthesis of corticotropin releasing hormone (CRH) and cortisol, which block GABA-ergic transmission and promote wakefulness [103]. In depression, similar mechanisms occur, feedback inhibition is changed in other ways, which causes the HPA axis to stay active for longer time. This dysregulation not only affects sleep, but also exacerbates depressive symptoms through reduced hippocampus neurogenesis and impaired stress adaptation [104]. In the context of FASD, dysregulation of the HPA axis is typically reported as a result of prenatal alcohol exposure, which disrupts the development of stress response mechanisms. This dysregulation could show up as reduced stress reactions or increased baseline cortisol levels, both of which can affect sleep patterns [73]. These effects are especially disturbing in the population with FASD, where anxiety and mood problems are common, making it even harder to get enough healthy sleep [105]. Knowing the bidirectional interactions among sleep, anxiety, and depression, we emphasize the need for combined therapeutic interventions. Targeting HPA axis normalization through pharmacological (e.g., glucocorticoid receptor modulators) and nonpharmacological (e.g., cognitive-behavioral therapy) interventions offers interesting directions for improving sleep and reducing psychiatric symptoms.

## 5. Limitations

Our study has several limitations that may affect the generalization of the findings. Although the sample size of 90 participants appears small, it is significant given the difficulties in recruiting children with FASD, making it a strength of this study. However, the specific background of the participants, mainly from foster or adoptive care settings, can limit the generalization of the results to the larger population of FASD. Therefore, we decided to include these data in the analysis as a potential confounder. Furthermore, relying on parent-reported measures, such as CDI 2:P, introduces potential bias because caregivers may overreport symptoms due to personal stress or a misinterpretation of the child’s behavior. Self-reported data from children, collected using the abbreviated version of CDI 2:SR, may also lack reliability due to cognitive and speech-related challenges common in FASD, and using the extended version may have produced different outcomes. Despite these limitations, the study has several strengths, including the use of validated psychometric tools, such as the CSHQ, STAIC and CDI 2, which improves the methodological rigor. It also focuses on an underexplored but clinically significant topic, offering important insights into the connections between emotional difficulties and sleep abnormalities in children with FASD. Key findings, such as the link between state anxiety and bedtime resistance or depressive symptoms and parasomnias, offer critical directions for future research. The results emphasize the need for targeted interventions that address sleep and emotional health in this population. More research is needed to examine these relationships and their implications for clinical practice.

## 6. Conclusions

In our study, we established that children with FASD have an increased tendency to depression and anxiety and that 71% of them report sleep problems. We also documented that state anxiety and depression correlate with the severity of sleep problems. Our findings implicate that sleep evaluation should be included in management of patients with FASD, and in cases where sleep problems are present, therapeutic options should be considered. Additionally, more research is needed to determine the bidirectional interactions between sleep and neurobehavioral symptoms in the FASD group.

## Figures and Tables

**Table 1:** Characteristics of the studied group of children diagnosed with FASD (*n* = 90). Data are shown as the number of children (the percentage of the valid number) or median (lower; upper quartile).

	Valid *n* (% of the Whole Studied Group)	Values in Studied Group
Age, years	90 (100)	10.5 (9; 12)
Male sex, *n* (%)	90 (100)	49 (54.4)
Type of care:	90 (100)	
Adoptive family, *n* (%)		46 (51.1)
Foster care, *n* (%)		23 (25.6)
Institutional care, *n* (%)		12 (13.3)
Biological family, *n* (%)		9 (10.0)
STAIC C-1	80 (88.9)	26 (23; 31)
STAIC C-1, centiles	80 (88.9)	45 (24; 72)
STAIC C-2	80 (88.9)	34 (29.5; 40.5)
STAIC C-2, centiles	79 (87.8)	71 (49; 88)
CDI 2:SR(S) TS	89 (98.9)	5 (3; 8)
CDI 2:SR(S) TS, centiles	89 (98.9)	72 (35; 79)
CDI 2:P TS	90 (100)	17.5 (13; 22)
CDI 2:P TS, centiles	90 (100)	84 (64; 94)
CDI 2:P EP	90 (100)	7.5 (4; 10)
CDI 2:P EP, centiles	90 (100)	85 (59; 94)
CDI 2:P FP	90 (100)	10 (7; 13)
CDI 2:P FP, centiles	90 (100)	59 (51; 67)

CDI 2:P—Children’s Depression Inventory 2: Parents; CDI 2:SR(S)—Children’s Depression Inventory 2: Self-Report (Short); EP—Emotional Problems; FP—Functional Problems; STAIC—State–Trait Anxiety Inventory for Children; TS—Total Score.

**Table 2:** Children’s Sleep Habits Questionnaire (CSHQ) Subscale and Total Scores in Children diagnosed with FASD (*n* = 90). Data are shown as the number of children (the percentage of the valid number) or median (lower; upper quartile).

	Valid *n* (% of the Entire Studied Group)	Values in the Studied Group
CSHQ Bedtime Resistance, Points	90 (100)	8 (6; 10)
CSHQ Sleep-onset Delay, Points	90 (100)	1 (1; 2)
CSHQ Sleep Duration, Points	90 (100)	4 (3; 5)
CSHQ Sleep Anxiety, Points	90 (100)	5 (4; 7)
CSHQ Night Wakings, Points	90 (100)	3 (3; 5)
CSHQ Parasomnias, Points	90 (100)	9 (7; 10)
CSHQ Sleep-Distorted Breathing, Points	90 (100)	3 (3; 5)
CSHQ Daytime Sleepiness, Points	90 (100)	12 (10; 15)
CSHQ Total Sleep Disturbance Score, Points	90 (100)	45 (41; 50)
CSHQ Total Sleep Disturbance Score > 41, *n* (%)	90 (100)	64 (71.1)

CSHQ—Children’s Sleep Habits Questionnaire.

**Table 3:** Significant correlations between STAIC results and CSHQ (Spearman’s correlation coefficients).

	Bedtime Resistance, Points	Sleep Anxiety, Points
STAIC C-1	r = 0.30; *p* = 0.008	r = 0.31; *p* = 0.005
STAIC C-1, centiles	r = 0.31; *p* = 0.005	r = 0.38; *p* < 0.001

NS—nonsignificant; STAIC—State–Trait Anxiety Inventory for Children.

**Table 4:** Significant correlations between CDI 2 results and CSHQ (Spearman correlation coefficients).

	Parasomnias, Points	Daytime Sleepiness, Points
CDI 2:SR(S) TS	r = −0.22; *p* = 0.037	NS
CDI 2:SR(S) TS, centiles	r = −0.26; *p* = 0.015	NS
CDI 2:P TS	NS	r = 0.32; *p* = 0.002
CDI 2:P TS, centiles	NS	r = 0.34; *p* = 0.001
CDI 2:P EP	r = 0.33; *p* = 0.002	r = 0.34; *p* < 0.001
CDI 2:P EP, centiles	NS	r = 0.21; *p* = 0.045
CDI 2:P FP	NS	NS
CDI 2:P FP, centiles	r = 0.23; *p* = 0.028	r = 0.37; *p* < 0.001

CDI 2:P—Children’s Depression Inventory 2: Parents; CDI 2:SR(S)—Children’s Depression Inventory 2: Self-Report (Short); CSHQ—Children’s Sleep Habits Questionnaire; EP—Emotional Problems; FP—Functional Problems; NS—nonsignificant; TS—Total score.

## Data Availability

Data are unavailable due to the privacy of patients.
